# Improvement of antioxidant capability by dietary *N*-acetyl cysteine supplementation alleviates bone loss induced by chronic heat stress in finisher broilers

**DOI:** 10.1186/s40104-024-01114-4

**Published:** 2024-12-01

**Authors:** Huaiyong Zhang, Herinda Pertiwi, Joris Michiels, Djoere Gaublomme, Maryam Majdeddin, Yuhuang Hou, Matthieu Boone, Dirk Elewaut, Iván Josipovic, Jeroen Degroote

**Affiliations:** 1 Department of Animal Sciences and Aquatic Ecology, Laboratory for Animal Nutrition and Animal Product Quality, Ghent University, Ghent, 9000 Belgium; 2 College of Animal Science and Technology, Henan Agricultural University, Zhengzhou, Henan 450046 China; 3 Unit Molecular Immunology and Inflammation, VIB Center for Inflammation Research, Ghent University and Department of Rheumatology, Ghent University Hospital, Ghent, 9000 Belgium; 4 Ghent University Centre for X-ray Tomography (UGCT), Ghent University, Ghent, 9000 Belgium; 5 Radiation Physics Research Group, Department of Physics and Astronomy, Ghent University, Ghent, 9000 Belgium

**Keywords:** Bone mass, Broilers, Heat stress, Intestine, *N*-Acetylcysteine, Oxidative stress

## Abstract

**Background:**

Heat stress (HS) incidence is associated with the accumulation of reactive substances, which might be associated with bone loss. *N*-Acetylcysteine (NAC) exhibits strong antioxidants due to its sulfhydryl group and being as the precursor for endogenous glutathione synthesis. Therefore, interplay between oxidative stress and bone turnover of broilers and the effects of dietary NAC inclusion on antioxidant capability and “gut-bone” axis were evaluated during chronic HS.

**Results:**

Implementing cyclic chronic HS (34 °C for 7 h/d) evoked reactive oxygen species excessive production and oxidant stress, which was accompanied by compromised tibia mass. The RNA-seq of proximal tibia also revealed the enrichment of oxidation–reduction process and inflammatory outbursts during HS. Although no notable alterations in the growth performance and cecal microbiota were found, the diet contained 2 g/kg NAC enhanced the antioxidant capability of heat-stressed broiler chickens by upregulating the expression of Nrf2 in the ileum, tibia, and bone marrow. Simultaneously, NAC tended to hinder NF-κB pathway activation and decreased the mRNA levels of the proinflammatory cytokines in both the ileum and bone marrow. As a result, NAC suppressed osteoclastogenesis and osteoclast activity, thereby increasing osteocyte-related gene expression. Furthermore, the inclusion of NAC tended to increase the ash content and density of the whole tibia, as well as improve cortical thickness and bone volume of the diaphysis.

**Conclusions:**

These findings HS-mediated outburst of oxidant stress accelerates bone resorption and negatively regulates the bone quality of tibia, which is inhibited by NAC in broilers.

**Graphical abstract:**

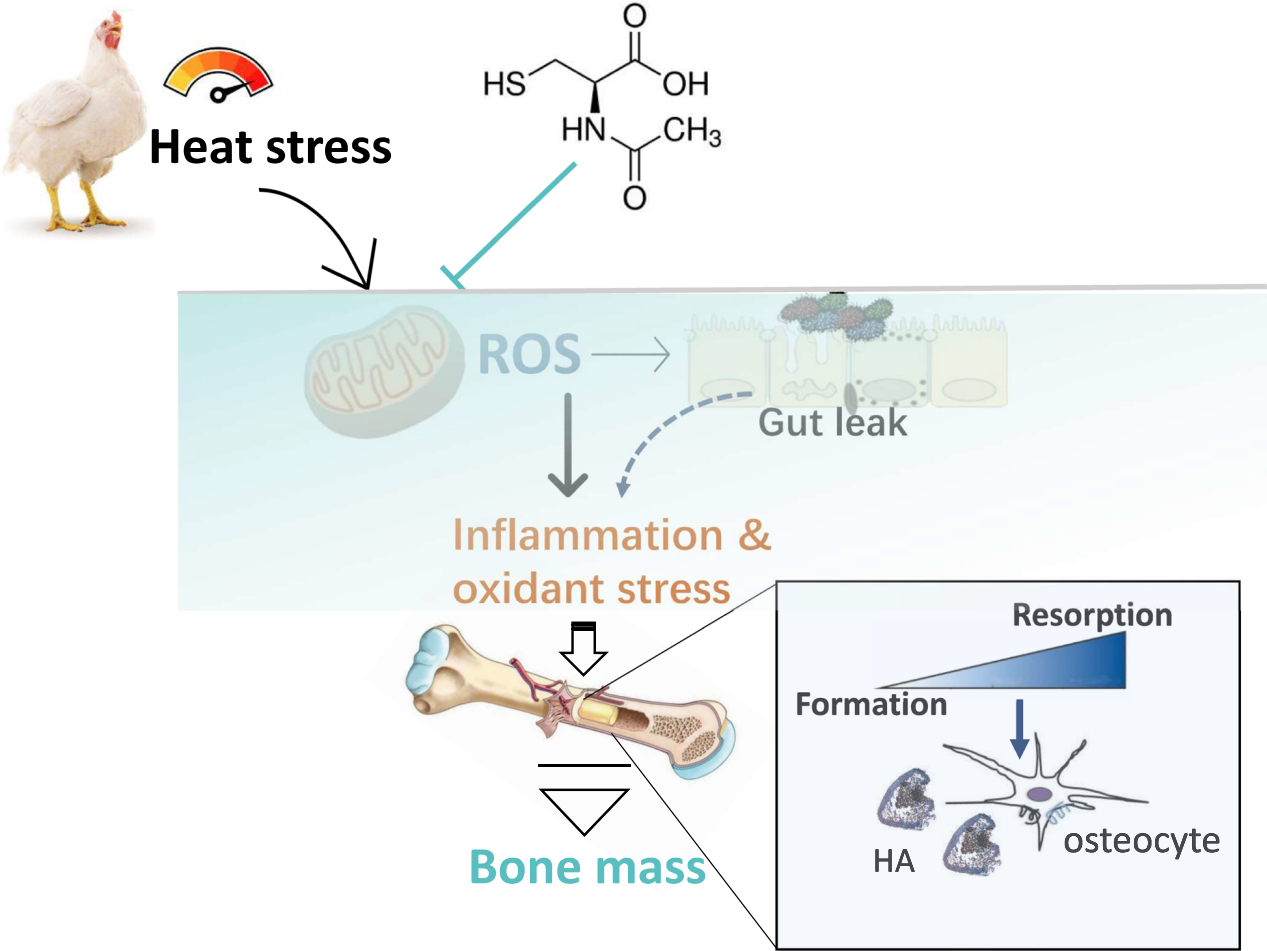

**Supplementary Information:**

The online version contains supplementary material available at 10.1186/s40104-024-01114-4.

## Introduction

Heat stress (HS) has been considered one of the main triggers of metabolism disorder, intestinal dysfunction, dysbiosis, bone abnormalities, etc., in the farmed animal industries [1]. Meat-type birds are highly sensitive to HS due to the cover of feathers, intensive metabolic heat production, and the lack of sweating [2]. Research has shown that heat exposure decreased the body weight (BW), feed intake (FI), and reduced feed conversion ratio of broilers, which is partly attributed to reduced intestinal absorption surface due to shortened villus height and deepened crypt depth under heat exposure conditions [3]. HS also resulted in tibia abnormalities characterized by decreased ash content and fracture load in broilers [4]. This will cause a reduction in mobility, which might be linked to pain and modified behavior [5]. However, the mechanism underlying leg problems and bone disorders caused by HS has not been fully elucidated thus far.

Along with deficient calcium (Ca) and phosphorus (P) absorption on account of reduced feed consumption [6], one of the potential reasons for poor bone quality caused by heat shock is associated with dysbiosis and impaired gut integrity [4]. It has been confirmed that HS could alter the gut microbiome and directly affect gut integrity, thereby inducing the activation of the innate immune system and inflammatory outbursts in laying hens [7] and broilers [4]. The production of inflammatory cytokines elicits osteoclastic bone resorption by upregulating receptor activator for nuclear factor-κB ligand (RANKL) expression and enhancing RANKL binding to its receptor RANK on osteoclast precursors [8], in which osteoprotegerin (OPG) secreted by osteoblast could also bind to RANK as a decoy receptor to hinder osteoclastogenesis [9]. This is why the RANKL-to-OPG ratio is usually deemed the key biomarker for evaluating osteoclast differentiation in practice. Moreover, nuclear factor-κB (NF-κB) signaling, a crucial inflammatory pathway, was activated in the process of RANKL-mediated osteoclastogenesis [10]. These findings highlight the interaction between intestinal barrier function and inflammatory reaction may be an important mediator for HS-induced bone loss. In fact, concomitant with the alteration in the immune system, the accumulation of reactive oxygen species (ROS) caused by HS interfered with bone remodeling, impairing bone quality [11]. Normally, the antioxidant defense system including superoxide dismutase (SOD), glutathione peroxidase (GSH-Px), and glutathione-*S*-transferases (GST) was initiated to maintain the balance between antioxidant and prooxidant substances, where the nuclear factor erythroid 2-related factor 2 (Nrf2)-mediated antioxidant related gene transcription exert an indispensable role [12]. Nevertheless, this balance can be compromised by elevated temperature and result in the production of excessive ROS, which adversely affects bone mass [11]. Clinical studies found that there is an inverse relationship between biomarkers of oxidative stress and bone mass [13]. Supplementation with an antioxidant diet was observed to reverse bone loss due to estrogen in postmenopausal subjects and ovariectomized mice [14, 15]. In heat-stressed broilers, the decreased ash content and fracture load of the tibia were also noticed, along with decreased SOD activity and increased plasma malondialdehyde (MDA) level, a lipid peroxidation marker in the ileum [4]. This evidence suggests that it is of great significance to develop feasible strategies to prevent bone loss caused by oxidant stress, especially in the broiler industry.

*N*-Acetyl cysteine (NAC, Fig. S1a) is an amino acid derivative and possesses excellent antioxidant properties through its sulfhydryl group to directly scavenge ROS and facilitate the synthesis of glutathione serving as a precursor [16]. Therefore, NAC has been increasingly applied to overcome the adverse effects of oxidative stress and inflammation in the livestock and poultry industry [17, 18]. For example, dietary supplementation of 0–2 g/kg of NAC linearly increased BW and reduced the ratio of feed consumption to body gain (F:G) in chronic cyclic heat-stressed finisher broilers [17]. The basal diet with 1 g/kg NAC was found to ameliorate ovarian damage by enhancing antioxidative capacity and depressing proinflammatory reaction in HS hens [19]. The protective roles of NAC on the intestinal barrier were also observed in lipopolysaccharide (LPS)-challenged piglets, evidenced by upregulated expression of claudin-1 and occludin in both jejunal and ileal mucosae [18]. Of note, a study on mice found that diet inclusion of 1 g/kg NAC improved bone microstructure and bone mineral density (BMD) of the femur in mice through elevating glutathione status [20]. More importantly, NAC is proven to protect against LPS-induced inflammation and estrogen deficiency that both cause bone loss via restraining bone resorption [20, 21]. It was pointed out that NAC could inhibit RANKL-induced ROS generation and hold up osteoclast differentiation in bone marrow cells [22]. In addition, NAC-promoting osteogenesis was visualized by upregulating the expression of runt-related transcription factor 2 (Runx2), Osterix, and bone morphogenic protein (BMP) in osteoblasts harvested from mouse calvaria [23]. However, whether NAC can attenuate bone loss of broilers and affect bone remodeling under the HS condition is poorly understood yet.

In the present study, the effects of cyclic chronic HS on tibial mass and oxidant status of broilers were explored, and we try to normalize antioxidant defenses using NAC and propose a protective role of NAC on the progression of HS-induced osteolysis in broilers, which sheds light on understanding the application of NAC in broiler production and treating bone diseases. Toward this end, histological analysis, transcriptome sequencing (RNA-seq), and 16S rRNA sequencing were performed to elucidate the potential molecular mechanisms underlying the relationship between HS or NAC and bone metabolism in the current study.

## Materials and methods

### Birds and diets

Day-old male Ross-308 male chickens were purchased from Vervaeke-Belavi (Tielt, Belgium), and subjected to the vaccination against Newcastle Disease and Infectious Bronchitis at 1 days of age, followed by the vaccination against Newcastle Disease at 18-day-old. They were reared in a climate-controlled facility, in which the initial temperature was set as 32 °C during 1–3 d and then gradually decreased to 22 °C by 21 d. The light schedule was 23 L:1 D and 18 L:6 D during 1–7 d and beyond, respectively. The basal diets include starter (1–14 d), grower (15–21 d), and finisher (22–39 d) phases were formulated to meet the Ross 308 nutrient requirements (Table 1). The dry matter (DM), crude protein (CP), ether extract, ash, Ca, and P of diets were analyzed as the standard procedure as shown in Table 2, which confirmed proper preparation of experimental diets.

**Table 1:** Composition and calculated nutrient content of experimental diets (as-fed basis)

Item	Starter (1–14 d)	Grower (15–21 d)	Finisher (22–35 d)
Ingredients, %			
Corn	55.83	59.75	63.49
Soybean meal	26.43	24.22	22.28
Toasted soybeans	12.00	10.00	8.00
Animal fat	0.000	0.697	1.443
Soybean oil	0.967	1.000	1.000
Monocalcium phosphate	1.167	0.962	0.901
Limestone	1.451	1.352	1.213
Sodium chloride	0.237	0.248	0.262
Sodium bicarbonate	0.341	0.352	0.182
Vitamin and mineral premix^a^	0.500	0.500	0.500
Choline chloride	0.100	0.100	0.100
Phytase 5000AL (500 FTU)	0.010	0.010	0.010
L-Lysine HCl	0.293	0.256	0.210
DL-Methionine	0.383	0.325	0.264
L-Threonine	0.150	0.120	0.087
L-Valine	0.140	0.104	0.064
Nutrient composition, %			
Dry matter	87.8	87.8	87.7
ME, MJ/kg	12.30	12.55	12.80
CP	21.6	20.0	18.5
Ether extract	6.07	6.47	6.89
Starch	37.5	39.8	42.0
Ash	6.01	5.55	5.08
Ca	0.86	0.78	0.71
P	0.62	0.56	0.53
Dig. P	0.43	0.39	0.37
Na + K − Cl, meq/100 g	27.0	25.5	22.0
C18:2	3.02	2.95	2.87
Lys	1.41	1.27	1.14
Dig. Lys	1.22	1.10	0.98
Dig. (Met + Cys)/Dig. Lys	0.75	0.75	0.75
Dig. Thr/Dig. Lys	0.67	0.67	0.67
Dig. Val/Dig. Lys	0.80	0.80	0.80

*ME* Metabolizable energy, *CP* Crude protein, *Ca* Calcium, *P* Phosphorus, *Dig.* Digestible, *Lys* Lysine, *Na* Sodium, *K* Potassium, *Cl* Chlorine, *Met* + *Cys* Methionine + Cysteine, *Thr* Threonine, *Val* Valine

^a^Premix providing per kg of diet: vitamin A (retinyl acetate), 10,000 IU; vitamin D_3_ (cholecalciferol), 2,500 IU (1–14 d) and 2,000 IU (15–39 d); vitamin E (DL-α-tocopherol acetate), 50 mg; vitamin K_3_ (menadione), 1.5 mg; vitamin B_1_ (thiamine), 2.0 mg; vitamin B_2_ (riboflavin), 7.5 mg; niacin, 35 mg; D-pantothenic acid, 12 mg; vitamin B_6_ (pyridoxine–HCl), 3.5 mg; vitamin B_12_ (cyanocobalamine), 20 µg; folic acid, 1.0 mg; biotin, 0.2 mg; choline chloride, 460 mg; Fe (FeSO_4_·H_2_O), 80 mg; Cu (CuSO_4_·5H_2_O), 12 mg; Zn (ZnO), 60 mg; Mn (MnO), 85; I (Ca(IO_3_)_2_), 0.8 mg; Co (Co_2_CO_3_(OH)_2_), 0.77 mg; Se (Na_2_O_3_Se), 0.15 mg

**Table 2:** Analysis of diet composition (as-fed basis)

Item	Starter	Grower	Finisher
	1–14 d	15–21 d	22–35 d
Dry matter, %	88.5	88.2	88.3
Crude protein, %	21.7	20.5	18.7
Ash, %	5.0	4.8	4.4
Calcium, %	0.80	0.83	0.68
Phosphorus, %	0.57	0.58	0.47
Ether extract, %	5.6	6.2	6.5
Vitamin D_3_, IU/kg	2,020	1,970	1,690

### Study design

To explore the effects of HS on tibia quality of broilers, broilers were individually weighed (the average BW was 0.478 ± 0.004 kg) at 15 d, and then divided into the chronic cyclic HS and thermoneutral room (constant 22 °C from 22 d onward). Each group had 7 replicates with 12 broilers per replicate. In the HS chamber, the HS was conducted by maintaining the temperature at 34 °C for 7 h daily with relative humidity (RH) between 50% to 60% and the rest of day at 26 °C from 22 to 39 d (Fig. S1b). In the thermoneutral room, a control group (Ctrl) was set up, in which these birds were allowed to feed the basal diets at libitum. Of note, to exclude the effects of HS on bone mass and oxidant properties due to the decreased feed intake, which was multiply confirmed in the previous studies [4, 24], a corresponding pair-feeding (PF) were carried out in thermoneutral room by measuring the daily FI of the HS group and providing the same amount the next day in three meals across the light hours of the day. BW and FI were recorded at 15, 21, and 39 d on a per-pen basis. On d 40, one chicken of average BW from each pen was selected from the HS unit at 4 h after starting HS and thermoneutral room for sampling. Birds were euthanized using an intravenous injection of 30 mg/kg of BW sodium pentobarbital. Blood was collected by the jugular vein with an 80 mm needle 22 G, and plasma was separated via centrifugation at 3,000 × *g* for 15 min. The proximal right tibiae were obtained, snap frozen in liquid nitrogen, and stored (−80 °C) for RNA-seq. The left tibiae were collected for bone property analysis.

The following experiment aimed to determine whether dietary supplementation with NAC could attenuate HS-induced bone loss. At d 21, birds with similar BW (979.7 ± 0.005 g) were randomly divided into either the HS group fed the basal diet, or a group fed the basal diet supplemented with 2.0 g/kg NAC (NAC group) until 35 d. Each group included 10 replicates of 12 birds per pen. The BW, FI, and the number of deaths and culling birds by pen were registered during the period of the trial. The body gain, feed conversion as the F:G, and survival proportions were calculated on a per-pen basis. At d 35, 4 h after starting HS, 2 birds were selected for sampling from each pen according to the average BW of the pen. The 1st bird was euthanized via intravenously injecting 30 mg/kg BW sodium pentobarbital. The whole blood was collected to separate the plasma referring to described above. The thymus, spleen, and bursa of Fabricius were dissected and weighed, and calculated as the relative weight of the organ. Segments of 1 cm length from the mid-ileum were dissected and stored in phosphate-buffered formaldehyde until analysis. The remaining ileum, from Meckel's diverticulum to the ileocecal junction (with 1 cm removed from the mid-ileum, totaling approximately 72 cm in length), was detach, mixed, and stored at −80 °C for antioxidant capability and gene expression analysis. The caeca contents were collected and snap-frozen in liquid nitrogen for the measurement of the microbiome and short-chain fatty acids (SCFAs). The proximal right tibia was excised and fixed using phosphate-buffered formaldehyde. The left tibia (the proximal end) and tibial marrow were collected and stored at −80 °C. The 2nd broiler was also euthanized using sodium pentobarbital, and the left tibia was removed and stored at −20 °C before being submitted to scans with micro-computed tomography (Micro-CT). The right tibia of each bird was harvested and cleaned of adherent tissue for the determination of bone properties.

### Heat stress model evaluation

Whether HS was successfully established would be evaluated via the respiratory rate that was called panting, the respiratory rate that was called panting, rectal temperature, and plasma heat shock protein (HSP70) content at 30 d. according to our previous description, a video contained at least 5 birds maintaining stand-still was over 1 min recorded using camera, the respiratory rates were counted when slowing down the playback speed. Moreover, 3 birds were randomly taken from each pen for Rectal temperature determination using a digital thermometer inserted to a minimum depth of 3 cm in the cloaca. Serum HSP70 was quantified using ELISA kit (CSBE11196Ch, Cusabio, Wuhan, China) according to the manufacturer’s instructions.

### Gait score

After sampling, birds remained subjected to chronic cyclic HS, and on d 36, 3 broilers were randomly selected from each pen and used for the evaluation of walking ability. Each bird was encouraged to walk approximately 10 m, and the score was recorded in this process according to a 6-point scale from 0 (completely normal) to 5 (unable to stand) as the method of Knowles et al. [25].

### Gut permeability determination

Gut permeability was evaluated based on fluorescein isothiocyanate dextran (FITC-d; 4 kDa, Sigma, Overijse, Belgium) leakage. Briefly, at d 36, one bird was selected randomly from each pen and given an oral gavage of FITC-d (4.16 mg/kg BW). One hour after FITC-d gavage, blood was collected and centrifuged at 3,000 × *g* for 15 min to obtain plasma. The fluorescence of plasma was measured using a fluorometer (Thermos Scientific, Merelbeke, Belgium) at 485 nm excitation and 530 nm emission. Levels of FITC-d were quantified from standard curves generated by the known levels of FITC-d and expressed as μg/mL of serum.

### Bone characteristics evaluation

After removing and cleaning adherent tissue, the length and perimeter of the right middle part of the tibia were measured by vernier calipers and flexible rulers, respectively. The fresh weight of bone was harvested, and the relative weight was calculated as the percentage of BW.

Tibia was subjected to biomechanical testing by the 3-point bending method with a texture analyzer (BFG 25,000 N force gauge, Mecmesin, Slinfold, UK). Loading proceeded at the mid-point of the tibia at a constant rate (12 mm/min) up to the breaking of the bone using a constant 50 kg load cell. The bone stiffness, ultimate load, and the area under curve (AUC) were obtained from the force-displacement data. Notably, the slope of the linear portion of the load-displacement curve was defined as stiffness.

The tibial fragment produced by biomechanical testing was collected, and the bone marrow cavities liquid was removed with absorbent paper. The bone volume (cm^3^) was measured based on the quantum of water overflowing due to the interposition of the tibia into a 100-mL measuring cylinder. Subsequently, the bone was immersed in ethyl ether to defat and air-dried. The free-fat weight was recorded, and the density (g/cm^3^) was calculated as bone fat-free weight (g) divided by tibial volume (cm^3^). These tibias were then ashed in a muffle furnace at 550 °C for 24 h. The ash content was expressed as the percentage of dry-defatted weight.

According to our previous description [4], the left tibia was cleaned of adherent tissue and dried at 65 °C to avoid the influence of the water content within the bone. The sample was scanned using the custom-designed micro-CT scanner HECTOR of the Ghent University Center for Xray Tomography (UGCT; www.ugct.ugent.be) at 22 mm isotropic voxel size with an X-ray source power of 130 kV and 10 W and integration time of 1,000 ms. An additional filter of 0.5 mm Al was used to reduce beam hardening effects. Images were collected from the total 37 mm in length of tibial mid-diaphysis and a region 4 mm in length of the proximal end of the tibia, starting 9 mm below the surface of the condyles, for the analysis of cortical and trabecular bone, respectively. The reconstruction of 2-dimensional was performed by using the in-house developed reconstruction platform. The images were analyzed by custom-written scripts in Image J (National Institutes of Health, USA). The bone volume to total volume ratio (BV/TV) and the average thickness of the structures were measured using the plugin from Bone J [26].

### Measurements of bone turnover markers in plasma

Plasma concentrations of Ca and P were determined using ammonium molybdate (Abbott Laboratories, Germany) and *o*-cresol phthalein (Sigma-Aldrich, Belgium), respectively. Plasma alkaline phosphatase (ALP), a bone formation marker, was measured by using a commercial kit from Abbott Laboratories (Wiesbaden, Germany). Bone resorption biomarkers type I procollagen C-terminal peptide CTX and TRAP levels were quantified by immunoassay purchased from Roche Diagnostics (Rotkreuz, Switzerland) and Sigma-Aldrich, Inc., (St. Louis, MO, USA), according to the instructions of the manufacturer.

### Ileum and tibia histological analysis

The fixed ileum was dehydrated, embedded, and sliced. Subsequently, the section was de-paraffinized, rehydrated, and stained with hematoxylin and eosin (H&E). Histopathological images were collected using a microscope equipped (Olympus, Aartselaar, Belgium). For each sample, at least 10 well-oriented villi units were selected to measure the villus height and crypt depth, and then the ratio of villus height to crypt depth was calculated.

In addition, the fixed proximal tibia was decalcified using 14% EDTA solution (pH 7.4) and embedded in paraffin. A 10-μm slice was prepared and subjected to tartrate-resistant acid phosphatase (TRAP) bone staining using the assay kit (Sigma-Aldrich, Overijse, Belgium). The osteoclasts were identified as TRAP-positive and the number of osteoclasts per bone surface (No. Oc/BS) was registered according to the previous method [27].

### Determination of GSH-Px, MDA, and ROS

The bone marrow was mixed with buffered aqueous extracts, containing 1% Triton X-100 phosphate buffer (pH 7; 50 mmol/L), homogenized, and centrifuged. Supernatant and plasma were used to detect total MDA content using the thiobarbituric acid reactive substances test [28]. In addition, the activity of GSH-Px in supernatant and plasma was determined according to the dynamical alteration in the oxidation of nicotinamide adenine dinucleotide phosphate and reaction time using Multi-Mode Microplate Readers at 340 nm [29], where the activity unit of GSH-Px was defined as the amount of sample (g) required to oxidize 1 μmol of 2,4-dinitrophenylhydrazine per minute at 25 °C. The content of ROS in plasma were measured by commercially available kits (Nanjing Jiancheng Bioengineering Institute, Nanjing, China).

### Cecal microbiome analysis

Genome DNA was extracted from each sample using the NucleoSpin® Soil Kit according to the manufacturer’s protocol (Macherey-Nagel, Düren, Germany) and modified to reduce potential PCR inhibitors (Eurofins Genomics, Konstanz, Germany). The V3–V4 hypervariable region of the 16S rRNA gene was amplified using universal primers (341F, 5′‐TACGGGAGGCAGCAG‐3′) and (805R, 5′‐CCAGGGTATCTAATCC‐3′). After purification, amplicons were sequenced on the Illumina Miseq platform (Illumina, San Diego, USA) with the V3 chemistry kit and 2 × 300 bp paired-end module (Eurofins Genomics, Konstanz, Germany). All reads passing the standard Illumina chastity filter were demultiplexed according to their index sequences. The raw reads were submitted to the DADA2 package in R (version 4.3.1). The obtained raw sequences were quality-trimmed and filtered. Amplicon sequence variants (ASVs) were inferred, and then forward and reverse reads were merged, and chimeras were removed following default settings or adjusted. Taxonomy was assigned using the SILVA database (v138.1). Low-count ASVs were removed with a threshold of 0.01% using the MicrobiotaProcess package (version 3.17) in R. The alpha diversity was evaluated by calculating the Chao1, Shannon, and Simpson index at the phylum level. The beta diversity was visualized using a principal coordinate analysis (PCoA) plot based on the Bray–Curtis distance, using the adonis2 function (vegan). Sequences generated in the current study have been deposited in the NCBI database.

### SCFAs content

An approximal 1 g of cecal sample was dissolved in 5.5 mL 10% formic acid, in which 0.5 mg ethyl butyric acid was added to serve as the internal standard. The supernatants were collected after centrifuging, and the total SCFAs, acetate, propionate, butyrate, iso-butyrate, valerate, and iso-valerate content were quantified by gas chromatography on a Shimadzu 2010 (Shimadzu Corporation, Netherlands) equipped with a flame ionization detector based on the previously method [30].

### Gene expression assays

Total RNA was extracted from the tibia, bone marrow, and ileal mucosa using the Trizol reagent (Sigma-Aldrich, Overijse, Belgium). After examination of the concentrations and quality of RNA, the complementary DNA (cDNA) was synthesized from 200 ng of total RNA using the PrimeScript™ RT Reagent Kit (RR037A, Takara, France). The mRNA expression was performed in the Lightcycler 480 II detection system (Roche) with Fast SYBR Green Master Mix (Takara). Primers were designed using online Primer 3 and presented in Table S1. Relative gene expression was determined by normalizing the expression of glyceraldehyde-3-phosphate dehydrogenase (*GAPDH*) and *β-actin* mRNA as previously described [31].

### Transcriptome analysis

Total RNA was isolated from the proximal tibia with TRIZOL reagent (Sigma-Aldrich, Belgium), and was subjected to the determination of the purity and integrity. Sequencing libraries were generated using the TruSeq RNA Sample Preparation Kit (Illumina, San Diego, CA, USA). The library preparations were sequenced on an Illumina NovaSeq 6000 platform (Novogene Biotechnology Co., Ltd., Beijing, China). Clean reads were collected by removing low-quality reads (Phred < 20), reads containing ploy-N and adapter from raw data. Reads were aligned to the *Gallus gallus* genome (GRCg6a) by using HISAT2 software. The differentially expressed genes (DEGs) between PF and HS group were identified by the HISAT2 software with log_2_|FC| ≥ 1 and *P* < 0.05. Gene ontology (GO) enrichment analysis and Gene Set Enrichment Analysis (GSEA) of the DEGs were implemented by the cluster Profiler R package.

### Statistical analysis

Data were analyzed using GraphPad Prism (GraphPad Software Inc., CA, USA). After checking the normal distribution and homogeneity of variances. An unpaired two-tailed *t*-test analysis was performed to elucidate the potential difference in the determined parameters. Survival curves were constructed using Kaplan-Meier method. Pearson's correlation analysis was performed to determine the correlation between tibia mass and serum oxidant indexes. For the microbial composition following 16S rRNA amplicon sequencing, statistical analyses were determined in R using the Microbiota Process package for community analysis (version 3.17). Data are presented as mean and standard deviation. *P* ≤ 0.05 and *P* < 0.10 was defined as statistically significant and trended, respectively.

## Results

### Cyclic chronic HS induced bone loss of finisher broilers

When compared to the Ctrl and PF groups, the birds subjected to heat exposure exhibited a notably increased (*P* < 0.05) rectal temperature and panting frequence (Fig. S1c and d). Moreover, the plasma HSP70 contents in heat-stressed birds were significantly elevated (*P* < 0.05) relative to the Ctrl and PF groups (Fig. S1e). This data indicates that the model of cyclic chronic HS was successfully estimated in this study.

**Fig. 1:**
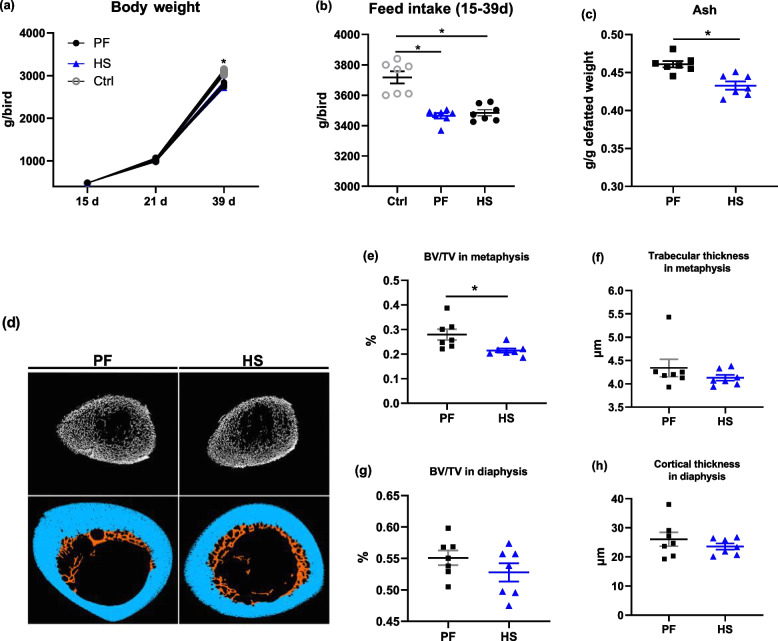
Effects of heat stress (HS) in broilers at d 39 on (**a**) body weight, (**b**) feed intake, and tibia mass, including (**c**) ash and (**d**–**h**) bone microstructure. (**d**) Micro-computed tomographic image of cortical bone from the tibia diaphysis of birds from pair feeding (PF) and HS groups illustrating tibia metaphysis (**e**) bone volume (BV/TV), (**f**) trabecular thickness, (**g**) BV/TV in diaphysis, and (**h**) cortical thickness (bule). * Denotes significant difference among Ctrl, PF, and HS at *P* ≤ 0.05

Data of growth performance show that implementing the chronic cyclic HS to broilers from 22 to 39 d significantly reduced the BW and food consumption during 15 to 39 d when compared to the Ctrl group but not the PF group (Fig. 1a and b). However, HS significantly reduced (*P* < 0.05) the ash content of tibia as compared to the PF group (Fig. 1c). Micro-CT analysis released a compromise in the microstructure of tibia in HS birds, i.e., lower BV/TV in metaphysis and diaphysis in HS compared with PF birds, which was accompanied by compared thickness of both trabecular and cortical bone in tibias (Fig. 1d–h).

### The poor bone quality induced by HS is associated with oxidant stress

**Fig. 2:**
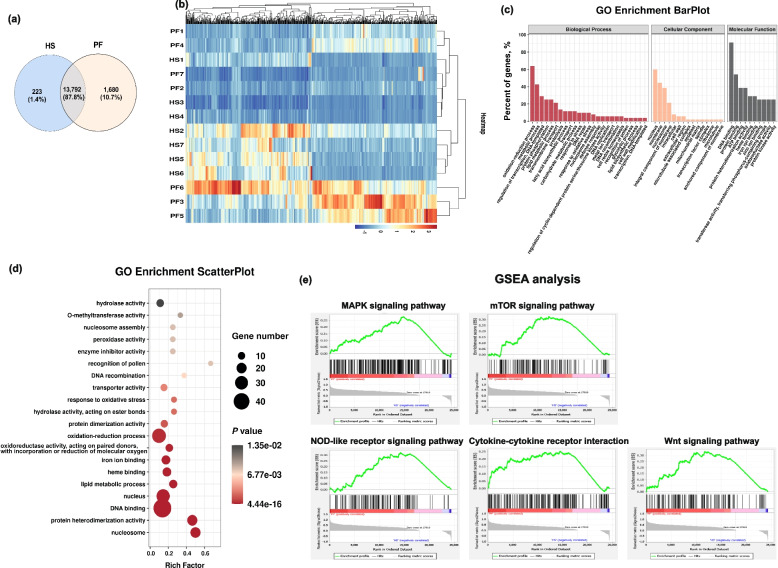
Transcriptome analysis for differentially expressed genes (DEGs) between heat stress (HS) and pair-fed (PF) groups. **a** Venn diagram showing the distinct and overlapping genes of the transcriptome. **b** Heatmap with hierarchical clustering analysis of DEGs of the transcriptome. The color scale indicates the mRNA expression levels. **c** and **d** Gene Ontology (GO) enrichment analysis. **e** Gene Set Enrichment Analysis (GSEA) of the DEGs. Genes with log_2_(FC) ≥ 1 and *P* < 0.05 were considered as significant DEGs

RNA-seq analysis was performed on the proximal tibia, and the expressions of matrix metallopeptidase (*MMP9*), Dickkopf WNT signaling pathway inhibitor 2 (*DKK2*), ETS variant 4 (*ETV4*), retinoic acid induced 2 (*RAI2*), and forkhead box C2 (*FOXC2*) in the transcriptome data were consistent with them in RT-qPCR results (Fig. S2), indicating the consistency and accuracy of the transcriptome data. Overall, 13,792 genes were detected in both the PF and HS groups, 223 and 1,680 genes were identified in the PF and HS groups, respectively (Fig. 2a). The notably alterations of the top 124 DEGs between the two groups were also manifested in the heatmap (Fig. 2b). GO analysis of differential genes showed that HS implementing was related to oxidation–reduction process and some metabolic processes including protein phosphorylation and lipid metabolic processes (Fig. 2c and d). Among the outcomes of GSEA analysis, the signaling pathway mitogen-activated protein kinases (MAPK), mammalian target of rapamycin (mTOR), NOD-like receptor, and Wnt, as well as cytokine-cytokine receptor strongly associated with heat exposure (Fig. 2e).

**Fig. 3:**
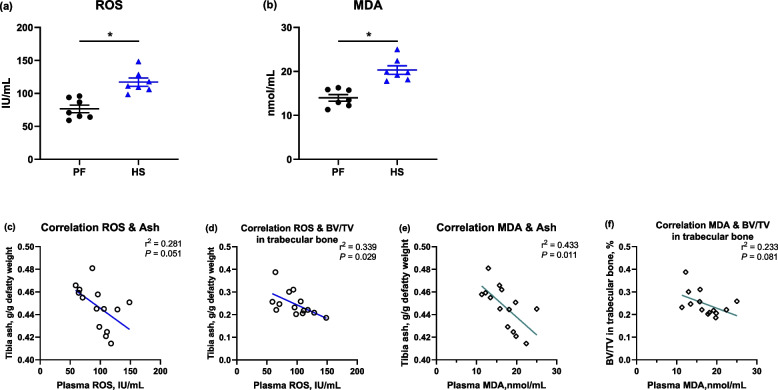
Analysis of the correlation between the reactive substances and bone mass of tibial bone in broiler. **a** Reactive oxygen species (ROS) and (**b**) malondialdehyde (MDA) in plasma were quantified, and (**c–f**) the correlation between them and tibia ash and bone volume (BV/TV) of trabecular bone were analyzed using Pearson’s correlation analysis. * Denotes significant difference between PF and HS. *P* ≤ 0.05 is defined as statistically significant

To confirm the relationship between oxidative stress and bone mass, the ROS and MDA levels of plasma were detected, and showed that heat exposure resulted in significantly increased (*P* < 0.05) MDA concentration and ROS levels in serum when compared to PF group (Fig. 3a and b). The production of ROS was negatively related with tibial ash content (*P* = 0.051) and BV/TV in trabecular bone (*P* < 0.05) based on the outcomes of Pearson's correlation analysis (Fig. 3c and d). Additionally, a negative correlation between serum MDA levels and bone mass, showed by both tibia ash and BV/TV in trabecular bone, was also noticed in this study (Fig. 3e and f).

### Dietary NAC inclusion improved gait score and tibia properties under HS

Dietary NAC treatment did not change the growth performance and mortality, showed by similar BW, gain, FI, F:G, and survival proportions when compared to the HS group (Fig. S3). Furthermore, as illustrated in Table 3, the diet containing NAC improved the walking ability of broilers under chronic HS conditions (*P* < 0.05). The positive roles exerted by NAC were not related to tibial growth, instructed by similar tibia length, perimeter, fresh weight, relative weight, and fat-free weight. Dietary supplementation of NAC increased the ash content (*P* = 0.055) and bone density (*P* = 0.063) of the whole tibia, as well as the cortical thickness (*P* = 0.055) and BV/TV of diaphysis (*P* = 0.038) compared with the HS group. Mechanical testing analysis showed that the HS and NAC groups exhibited no obvious alterations in the tibia stiffness, ultimate strength, and AUC (*P* < 0.05).

**Table 3:** Effect of dietary *N*-acetyl cysteine (NAC) supplementation on gait score (d 36) and tibia bone characteristics (d 35) of broilers

Item	HS	NAC	*P*-value
Gait score	2.57 ± 0.61	1.93 ± 0.54^*^	0.024
Bone growth			
Length, mm	10.69 ± 0.41	10.66 ± 0.37	0.867
Perimeter, mm	2.98 ± 0.19	2.91 ± 0.33	0.574
Fresh weight, g	17.43 ± 1.38	17.26 ± 1.91	0.826
Relative weight, g/kg body weight	6.64 ± 0.43	6.71 ± 0.82	0.799
Fat-free weight, g	6.98 ± 0.55	6.87 ± 0.84	0.718
Bone mass			
Ash, % Fat-free weight	41.09 ± 1.14	42.49 ± 1.80	0.055
Density, g/cm^3^	0.46 ± 0.02	0.47 ± 0.01	0.063
Cortical thickness in diaphysis, μm	13.26 ± 2.83	15.97 ± 2.46	0.055
BV/TV in diaphysis, %	45.10 ± 2.43	47.62 ± 2.62^*^	0.038
Trabecular thickness in metaphysis, μm	3.92 ± 0.33	3.83 ± 0.27	0.532
BV/TV in metaphysis, %	22.17 ± 2.05	21.06 ± 2.10	0.246
Bone mechanical properties			
Stiffness, N/mm	222.55 ± 54.90	208.92 ± 67.35	0.626
Ultimate strength, N	366.15 ± 41.00	329.40 ± 89.50	0.259
AUC, N × mm	546.39 ± 111.26	559.82 ± 161.71	0.831

Data represents means with standard deviation

*BV/TV* Bone volume/Total volume, *AUC* Area under the load-displacement curve

^*^Denotes significant difference between HS and NAC at *P* < 0.05

### Supplementation with NAC suppressed bone resorption

**Fig. 4:**
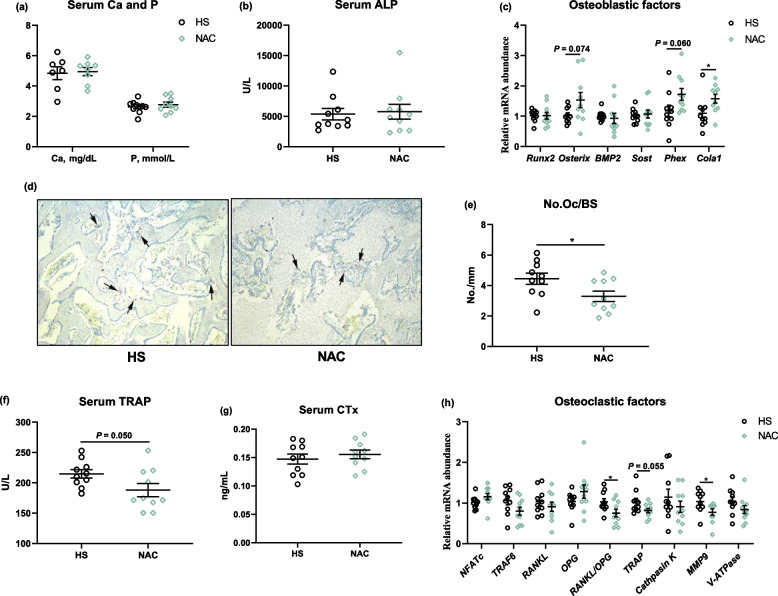
Serum calcium (Ca) and phosphorus (P) (**a**) and bone turnover of heat-stress broilers fed diet with NAC. (**b**) Plasma bone formation biomarker alkaline phosphatase activity (ALP), (**c**) The mRNA expression of osteoblastic factors; (**d**) Tartrate-resistant acid phosphatase (TRAP) staining and (**e**) the number of TRAP-positive cells (osteoclasts) were calculated in proximal tibia sections. Plasma bone resorption biomarkers including (**f**) TRAP and (**g**) C-terminal cross-linked telopeptide of type I collagen (CTx) levels, (**h**) transcription levels of osteoclastic factors. All the results were shown as mean ± standard deviation. An unpaired two-tailed *t*-test analysis was used to evaluate differences. ^*^P ≤ 0.05. Runx2, runt related transcription factor 2; BMP, bone morphogenetic protein; Sost, Sclerostin; Phex, phosphate regulating endopeptidase homolog x-linked; Col1a1, collagen type I alpha 1 chain; NFATC1, nuclear factor of activated T-cells 1; TRAF6, TNF receptor associated factor 6; OPG, osteoprotegerin; RANKL, Receptor activator of nuclear factor-κ B ligand; MMP9, matrix metallopeptidase 9; V-ATPase, V-type proton ATPase

Figure 4a showed that dietary NAC supplementation did not significantly affect the content of Ca and P in plasma. No obvious differences were also seen in plasma ALP activity, following NAC treatment in the HS broilers (Fig. 3b). The osteoblastic gene including *Osterix* (*P* = 0.074), phosphate regulating endopeptidase homolog x-linked (*Phex, P* = 0.060), and collagen type I alpha 1 chain (*Cola1*,* P* < 0.05) were upregulated by the dietary NAC addition when compared to the HS group (Fig. 4c). As far as bone resorption is concerned, lower TRAP-positive cells (osteoclast) were observed in the proximal tibia region in the broilers fed the NAC diet than those fed the basal diet (Fig. 3d and e). Accordingly, in comparison with the HS group, the NAC-treated birds showed remarkably decreased TRAP activity (*P* = 0.05) but not Ctx level in plasma (Fig. 4f and g). Although the diet with NAC did not obviously alter the expression of *RANKL* and *OPG*, it significantly reduced (*P* < 0.05) the ratio of *RANKL* to *OPG* in the proximal tibia as compared to the HS group (Fig. 4h). After NAC treatment, the transcriptions of *TRAP* (*P* = 0.055) and *MMP9* (*P* < 0.05) were also decreased than that in the HS group (Fig. 4f).

### NAC administration enhanced the antioxidant properties and inflammation of birds

**Fig. 5:**
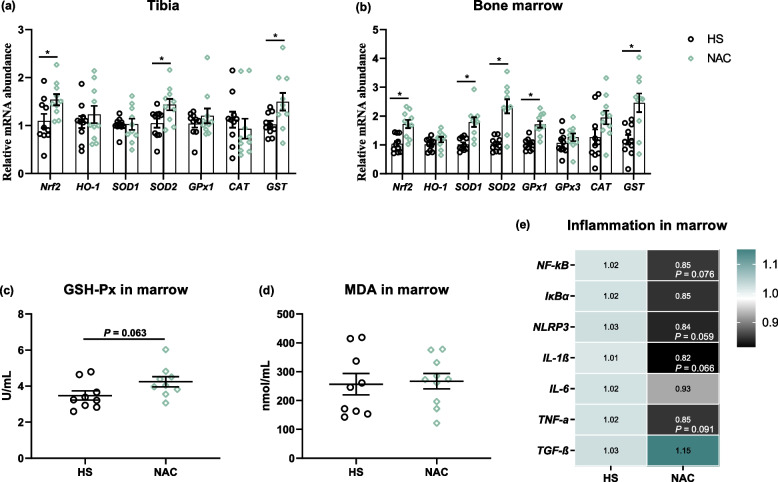
Effects of NAC on the antioxidant properties and inflammatory reaction of tibia and bone marrow. **a** and **b** The gene expression of nuclear factor, erythroid 2 like-2 (*Nfr2*), heme oxygenase-1 (*HO-1*), superoxide dismutase (*SOD*), glutathione peroxidase (*GPx*), catalase (*CAT*), and glutathione *S*-transferase theta 1 (*GST*) in the tibia and bone marrow. (**c**) Glutathione peroxidase (GSH-Px) activity and (**d**) malondialdehyde (MDA) level in the bone marrow. **e** The mRNA abundances of nuclear factor-kappa B (*NF-kB*), IκB alpha (*IκBα*), NLR family pyrin domain containing 3 (*NLRP3*), interleukin (*IL*)-*1β*, *IL-6*, tumor necrosis factor alpha (*TNF-α*), and transforming growth factor beta (*TGF-β*) in the bone marrow. All the results were shown as mean ± standard deviation. An unpaired two-tailed *t*-test analysis was used to evaluate differences. ^*^P ≤ 0.05

Considering the effects of dietary NAC on the antioxidant properties and inflammation of bone. As depicted in Fig. 5a, the broilers fed the NAC diet possessed higher mRNA levels of *Nrf2*, *SOD2*, and *GST* in the proximal tibia than those who received the basal diet (*P* < 0.05). In bone marrow, the upregulations in *Nrf2*, *SOD1*, *SOD2*, *GPx1* (encoding GSH-Px), and *GST* expression were also observed in the NAC group when compared to the HS group (*P* < 0.05, Fig. 4b). Administration of NAC increased the levels of GSH-Px (*P* = 0.063) relative to the HS group, which was accompanied by an indifferent MDA content in bone marrow (Fig. 5c and d). As for the inflammatory status of bone marrow, the NAC-treated group had a lower mRNA richness of *NF-κB*, interleukin (*IL*)-*1β*, NLR family pyrin domain containing 3 (*NLRP3*), and tumor necrosis factor alpha (*TNF-α*) compared to the HS chickens (Fig. 5e).

**Fig. 6:**
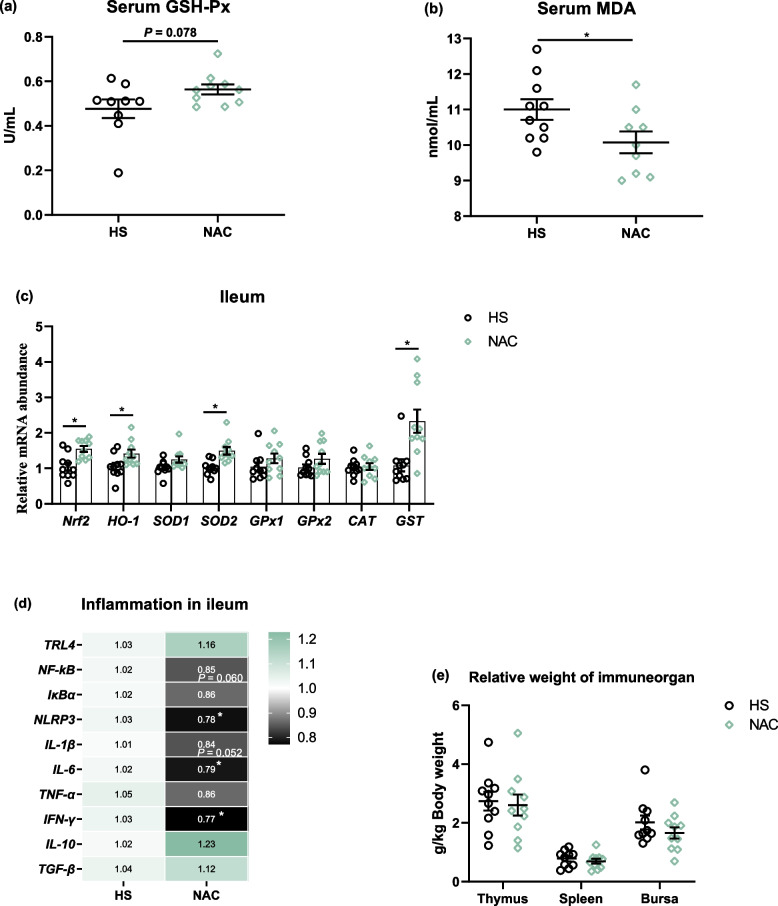
NAC enhanced the antioxidant capability and reduced inflammation of ileum in heat-stressed broilers. **a** and **b** The levels of glutathione peroxidase (GSH-Px) and malondialdehyde (MDA) in plasma. **c** The mRNA abundances of nuclear factor, erythroid 2 like-2 (*Nfr2*), heme oxygenase-1 (*HO-1*), superoxide dismutase (*SOD*), glutathione peroxidase (*GPx*), catalase (*CAT*), and glutathione *S*-transferase theta 1 (*GST*) in the ileum. **d** Ileal mRNA abundances of toll like receptor 4 (*TRL4*), nuclear factor-kappa B (*NF-kB*), IκB alpha (*IκBα*), NLR family pyrin domain containing 3 (*NLRP3*), Interleukin (*IL*)-*1β*, IL-6, tumor necrosis factor alpha (*TNF-α*), Interferon-γ (*IFN-γ*), *IL-10*, transforming growth factor beta (*TGF-β*). **e** Relative weight of thymus, spleen, and bursa. All the results were shown as mean ± standard deviation. An unpaired two-tailed *t*-test analysis was used to evaluate differences. ^*^P ≤ 0.05

The examination of serum obtained from the NAC broilers showed a trend for increased activity of GSH-Px (*P* = 0.078) and a significant decrease (*P* < 0.05) in plasma MDA levels when compared to the HS group (Fig. 6a and b). RT-PCR data revealed that the NAC-treated group displayed a notably up-regulated expression of *Nrf2*, heme oxygenase 1 (*HO-1*), *SOD2*, and *GST* in the ileal mucosa as compared to the HS group (Fig. 6c). Detection of the inflammatory status showed that diet NAC inclusion tended to decrease the mRNA abundances of *NF-κB* (*P* = 0.060) and *IL*-*1β* (*P* = 0.052), and remarkably upregulated the transcription of *NLRP3*, *IL-6*, and interferon (*IFN*)*-γ* relative to the HS group (Fig. 6d). There were no apparent differences in terms of the relative weight of the thymus, spleen, and bursa between HS and NAC birds (Fig. 6e).

### Dietary NAC treatment promoted intestinal integrity, but it failed to alter the cecal microbiota composition and the production of SCFAs

**Fig. 7:**
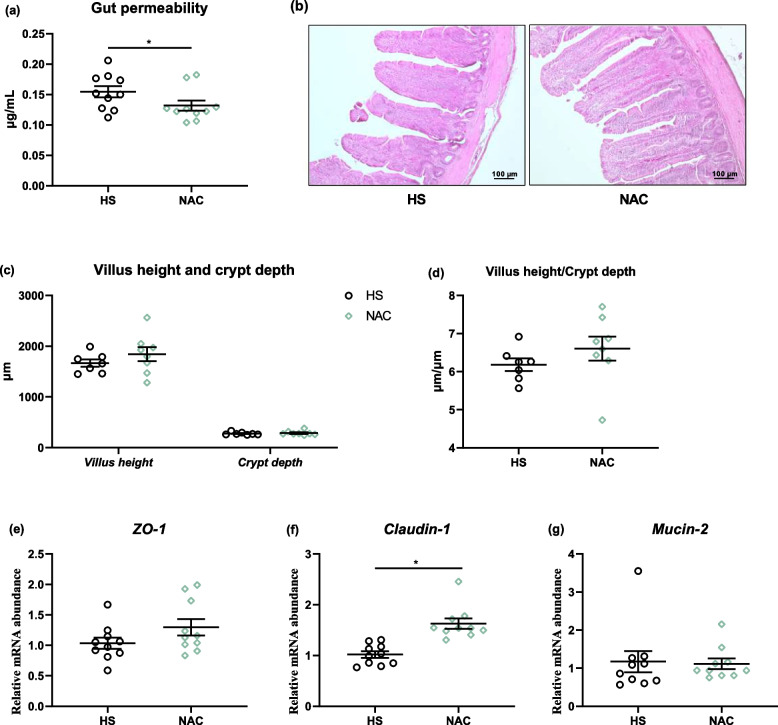
Dietary NAC improved intestinal integrity of heat-stressed broilers at d 35. **a** Gut permeability was directly evaluated via fluorescein isothiocyanate dextran (FITC-d); **b** Hematoxylin and eosin (H&E) staining (× 100), **c** villus height and crypt depth, and (**d**) their ratio in ileum. The mRNA abundance of (**e**) zonula occludens-1 (*ZO-1*), (**f**) *claudin-1* and (**g**) *mucin-2*. All the results were shown as mean ± standard deviation. An unpaired two-tailed *t*-test analysis was used to evaluate differences. ^*^P ≤ 0.05

In comparison to the HS group, there was a notably lower FITC-d concentration in plasma in response to NAC supplementation (*P* < 0.05, Fig. 7a), whereas the NAC administration not significantly increase the villus height and its ratio to crypt depth (Fig. 7b–d). The outcomes of tight junction proteins (TJPs) expression showed that NAC manipulation remarkably upregulated the mRNA level of *claudin-1* relative to the HS group (*P* < 0.05), which was accompanied by nondifferent mRNA abundances of zonula occluden-1 (*ZO-1*) and *mucin-2* (Fig. 7e–g).

**Fig. 8:**
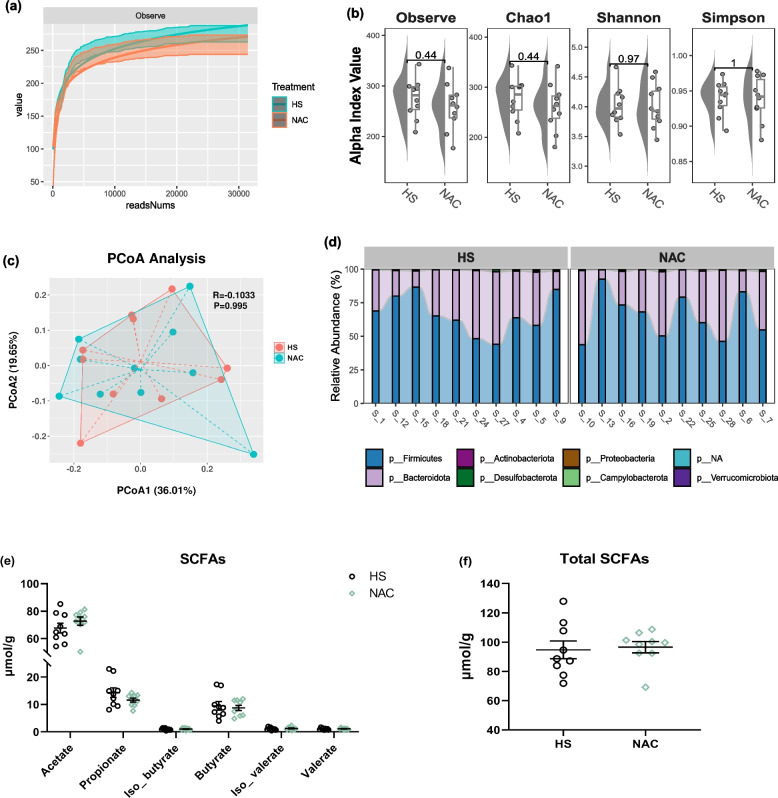
Cecal microbiota composition and short-chain fatty acids (SCFAs) response to NAC treatment in heat-stressed broilers. **a** The rarefaction curve, (**b**) Chao1, Shannon, and Simpson indexes were used to assess α-diversity at phylum level, (**c**) principal coordinate analysis plot (PCoA) of cecal microbiome diversity at phylum level based on Bray-Curtis, (**d**) bacterial communities at phylum level, (**e** and **f**) SCFA content in cecal chyme. *P* ≤ 0.05 was defined statistically significant

According to Fig. 8a, the rarefaction curve reflecting the diversity of species in the sample shows that the amount of sequencing data is reasonable in this study. However, dietary NAC failed to affect the Chao1, Shannon, and Simpson indexes when compared to the HS group (Fig. 8b). Moreover, the samples in the NAC group did not form a distinct cluster from those in the HS group (Fig. 8c). At the phylum level, the Firmicutes and Bacteroidetes phyla were the dominant in the cecal microbiota of broilers, and the HS and NAC groups displayed no alteration in the composition of microbiota (Fig. 8d). Regarding SCFA contents, the broilers that received the NAC diet did not exhibit different levels of total and individual SCFA in the cecal chyme as compared to HS birds (*P* > 0.05; Fig. 8e and f).

## Discussion

High ambient temperature-induced HS is a serious condition, with gut leak, dysbiosis, bone loss, and multi-organ dysfunction that cause detrimental effects on livestock and poultry [1, 4], especially for broiler chickens. Exposure of broilers to high room temperature has been confirmed to impair gut integrity and magnify inflammation-induced bone resorption, which result in poor bone quality [4, 32]. Similarly, the deleterious effects of HS on bone properties were also verified in the present study by decreased ash content and BV/TV in tibia, which were negatively correlated with the production of MDA and ROS, implying that the oxidant stress elicited by heat exposure might be one of the evocators for bone loss. As a well-known antioxidant, supplementation with NAC might reverse heat induced alterations in tibia quality and improve walking ability in finisher broilers. This might be related to the decrease in the bone resorption of tibias through enhancing the gut integrity, antioxidant capacity, and anti-inflammatory status thanks to the dietary supplementation of NAC in finisher broilers suffering from chronic HS.

It is well-established that HS is associated with bone disorders in domestic birds, including broilers [32, 33], laying hens [34], and turkeys [35]. One of main reasons induced bone loss is the deficient nutrient absorption, especially Ca and P, due to the reduced feed consumption under condition of heat exposure [8]. Thereby, the pair feeding was included to decipher whether HS effects are solely because of reduced FI in this study, and the indifferent FI during 15–35 d was observed between PF and HS groups. Compared to the PF group, results of bone tibia properties demonstrate that the tibiae from HS-birds displayed a decrease in bone mass, evidenced by lower ash content and BV/TV in metaphysis in the current trial. In the outcomes of RNA-seq of proximal tibia, the GO and DEGs enrichment analysis revealed that the changed tibial characteristics are probably associated with the alterations in oxidation–reduction process and some metabolic processes including protein phosphorylation and lipid metabolic processes, which might involve in the signaling pathway MAPK, mTOR, NOD-like receptor, and cytokine-cytokine receptor. It was confirmed that MAPK signaling and NF-κB pathway serving as the upstream and downstream of mTOR, respectively, play key roles in intestinal inflammation and oxidative stress [36]. Moreover, Wnt signaling is closely linked to bone homeostasis by regulating the activity of osteoblast and osteoclast [37]. In general terms, osteoblasts initiate bone formation, whereas osteoclasts take charge of bone resorption during bone modeling and remodeling. Combining the deleterious effects of oxidative stress in bone remodeling [11], it is possible that oxidative stress induced by HS interfered with bone remodeling, impairing bone quality of boilers. Accordingly, in the current study, MDA and ROS was quantified and showed a negative correlation between them and bone mass. Indeed, there was an inverse and positive relationship between biomarkers of oxidative stress and bone mass in clinical trials [13]. Data from postmenopausal subjects and ovariectomized mice showed that feeding an antioxidant diet could reverse bone loss due to estrogen [14, 15]. In heat-stressed broilers, we recently observed that heat shock led to deterioration of ash content and fracture load of the tibia, which was accompanied by increased MDA level in serum and decreased SOD activity in the ileum [4], implying dietary supplements with NAC, a powerful antioxidant, could potentially be useful to bone health. It was reported that a diet with 1 g/kg NAC elevated the bone volume, the thickness of the trabecular, and BMD of the femur in mice receiving a high-fat diet through elevating glutathione status [20]. The suppression effects of NAC on bone loss were also found in LPS-induced and ovariectomized mice [20, 21]. In this study, NAC manipulation increased tibial ash, density, as well as the bone volume and cortical thickness of diaphysis, but did not alter bone dimension, weight, and mechanical properties. This suggests that dietary NAC can improve tibia mass of heat-stressed broiler chickens by inhibiting oxidative stress. It is worth stressing that no difference was also observed in terms of bone dimension between the control and NAC group in mice [20]. In a rat model of kidney disease-mineral bone disorder, NAC did not alter the bone mechanical properties of proximal tibia [38].

To provide direct evidence of how the dietary NAC addition affects bone metabolism, bone formation and bone resorption were assessed biochemically and histologically. Multiply studies have been conducted to clarify that excessive oxidative stress hinders bone formation by delaying the proliferation and differentiation of osteoblasts [39–41]. It was demonstrated that oxidative stress induced by H_2_O_2_ resulted in osteoblastic apoptosis and osteogenic function in MC3T3-E1 osteoblast- like cell line [39] and rat bone marrow-derived osteoblastic cells [40]. Oxidative stress interferes with osteoblast involved in the activation of the Nrf2/HO-1 signaling pathway, which might depend on Runx2 transcription [42]. The suppression of Runx2 and the corresponding downregulation of ALP and osteocalcin could be responsible for the reduction in osteoblast differentiation and mineralization in the model of H_2_O_2_-mediated oxidant stress [39]. Also, the differentiation markers of osteoblasts including ALP and collagen I were significantly reduced by H_2_O_2_ exposure on primary rabbit bone marrow mesenchymal stem cells (BMSCs) [43]. A study on MC3T3-E1 found that pretreatment with NAC could restore the decrease in mineralization and ALP activity induced by H_2_O_2_ [39]. The enhanced influences of NAC on osteoblastic differentiation were also manifested by upregulated expression of *Runx2*, *Osterix*, and *BMP* in mouse calvaria cells [23]. One of the potential motivators of NAC to osteoblastic function probably is associated with the glutathione status [23], which was further supported by the published studies saying that NAC restored H_2_O_2_-induced reduction of Cola1 and osteocalcin gene expression, as well as osteoblastic mineralization was linked to an increased level of cellular glutathione and a decreased level of ROS in an osteoblastic cell model [40]. In the current study, the expression of osteogenic genes including *Runx2*, *Osterix*, and *BMP2* were nondiscriminatory between HS and NAC groups, while the transcription of *Phex* and *Cola1*, both the osteocyte markers, were increased following NAC supplementation. Considering the comparable ALP levels in plasma, these findings suggested that the improvement of tibia mass in NAC birds might not be associated with change in bone formation, whereas the supplementation of NAC probably promotes the transformation of osteoblast into osteocyte. At this point, NAC has been demonstrated to serve as an osteogenic- enhancing molecule, not to induce the translation of BMSCs into osteoblast progenitor cells [44].

Under the oxidant stress condition, it was shown that ROS directly evokes and/or serves as a signal mediator in the RANKL signaling cascade to induce osteoclast formation and activity, initiating bone resorption [45, 46]. Restoring redox balance by manipulating antioxidants (such as NAC, glutathione, and ascorbate) could depress the activity and attenuate estrogen deficiency-induced bone loss in mice [47]. In the present study, dietary NAC inclusion improved the antioxidant capacity of the tibia and bone marrow, instructed by upregulated expression of *SOD2* and *GST* in both tibia and marrow, as well as the activity of GSH-Px in bone marrow. At the same time, our results indicate that dietary NAC treatment reduced osteoclast number in the proximal tibia and plasma TRAP activity, as well as downregulated the osteoclastic gene expression of *TRAP* and *MMP9* in tibia and the ratio of *RANKL* to *OPG*, suggesting the antioxidant restoration owing to NAC could inhibit the difference and activity of osteoclast and depress bone resorption. Consistently, it was reported that dietary NAC addition mitigated adiposity-induced bone loss by decreasing oxidant stress-mediated bone resorption in mice [20]. The powerful antioxidant effects of NAC on reducing ROS formation in osteoclasts was noticed in LPS-challenged mice [21]. A previous study also found that NAC could restrain RANKL-induced ROS production and cut off osteoclast differentiation in bone marrow cells [22]. Additionally, the upregulated Nrf2 gene expression in both tibia and bone marrow in the current study implied that Nrf2 signaling pathways are possibly responsible for mediating NAC effects. Specificity, following the accumulation of ROS under heat stroke, cells attempt to restore the redox balance by enhancing its antioxidant capability, in which Nrf2, a redox-sensitive transcription factor, could transfer to the nucleus from the cytoplasm and stimulate antioxidant-related gene transcription [12]. Emerging evidence manifested that Nrf2 can weaken osteoclast differentiation during the process of RANKL acting on precursor osteoclasts [48]. Nrf2 knockout in bone marrow-derived macrophages accelerated osteoclastogenesis and resorption pits in dentine disks [49]. Moreover, the suppression of osteoclastic differentiation is probably attributed to the anti-inflammatory action of NAC based on published research [50]. NF-κB signaling pathway is regarded as a key molecule in RANKL-mediated osteoclastogenesis and ROS production, once NF-κB was activated, the osteoclastic gene expressions were enhanced, eventually leading to the formation and differentiation of osteoclast [10]. In this study, NAC treatment tended to inhibit the transcription of *NF-κB*, *NLRP3*, *IL-1β*, and *TNF-α* in bone marrow, demonstrating that NAC-decreased osteoclastogenesis might be associated with the downregulated NF-κB pathway.

Of note, the suppressed role of NAC in bone resorption might be related to the enhanced intestinal integrity, supported by the fact that higher intestinal permeability observed in diseases, such as inflammatory bowel disease, is correlated with bone loss [51]. Normalizing intestinal integrity caused by *Salmonella* using MDY, a known intestinal protector, could protect the loss of trabecular bone in broilers [52]. Studies have fostered a well understanding that the intestine is susceptible to ambient stress [53]. Data from our recent research revealed that pathological damage of the ileum was manifested in broilers following chronic HS, including depressed expressions of TJPs and increased gut leak [4]. In this study, the feeding of NAC diets upregulated the transcription of *claudin-1* in the prophylactic supplementation of 20 mmol/L NAC in a liquid diet reversed the radiation-induced decrease of TJPs such as ZO-1 and E-cadherin in mouse colon [54]. Similarly, feeding of 500 mg/kg NAC in diet enhanced the intestinal integrity of LPS-challenged piglets, as indicated by upregulated expression of claudin-1 and occludin in both jejunal and ileal mucosae, as well as the activity of diamine oxidase, a marker of intestinal integrity [18]. One of the manners by which HS impaired intestinal barrier is through oxidative stress [55]. To maximize heat dissipation under heat exposure, the redistribution of blood toward peripheral organs might trigger hyperthermia, hypoxia, or inflammation of the intestine, which can induce oxidative stress [56]. As a thiol-containing antioxidant, NAC exhibits an excellent antioxidant property by directly neutralizing free radicals [16]. NAC also enhances the status of cellular glutathione via increasing GST transcription to contribute to its antioxidant properties [16, 57]. In this regard, the protective roles of NAC in the intestine against oxidative stress were noticed, evidenced by reduced content of free radicals and lipid peroxidation, as well as promoted activities of antioxidant enzymes in the gut [58]. Analogous to previous studies [39, 59, 60], the current study also confirmed that NAC reduced the levels of serum MDA, improved the serum GSH-Px activity, and the expression of *Nrf2*, *HO-1*, *SOD2*, and *GST* in the ileum of HS-challenged broilers, indicating that dietary NAC supplementation enhanced intestinal barrier probably attributed to the improvement in antioxidants capability. In addition, it is a remarkable fact that the overproduction of ROS under HS conditions also resulted in an NF-κB-mediated inflammatory response and induced the release of proinflammatory cytokines [61]. It was reported that the elevated intestinal IL-1β, IL-6, and TNF-α levels were linked to an increased intestinal permeability by reducing occludin and ZO-1 protein levels [62]. The downregulated expressions of inflammatory cytokines such as TNF-α and IL-1β were noticed to recover the colonic ZO-1 protein abundance in the colitis mice [63]. Inhibition of NF-κB activation can protect the intestinal integrity of heat-stressed broilers by stimulating the abundance of TJPs [64]. Moreover, NAC could inhibit the NLRP3/IL‑1β signaling pathway, a key inflammasome, to reduce the inflammatory response in MSCs treated by LPS [60]. Collectively, in this study, it was proposed that NAC attenuates the oxidant stress and inflammation in the intestine of HS birds, and it would benefit the restoration of intestinal integrity of broilers.

In addition, the alteration in gut microbiota also plays a key role in bone metabolism. It was noticed that germ-free mice characterized by higher bone mass were showing a reduced number of osteoclasts and lower level of IL-6, RANKL, TNF-α, and CD4^+^T cells in bone [65, 66], whereas colonization with gut microbiota from conventionally raised mice normalized these features were [66], indicating that the interaction between gut microbiota and the immune system may play a significant role in bone metabolism [8]. An increase in cecal *Firmicutes* proportion was found to depress the expressions of proinflammatory cytokines in bone marrow, and consequently decrease bone resorption and improve tibia quality in meat ducks [65]. Study on broilers suggests that the changed proportion of Firmicutes and Bacteroidetes owing to heat shock was associated with inflammation outburst in bone marrow, resulting in osteoclastogenesis and bone loss. Accordingly, intervening gut microbiota might provide promising strategies for restoring bone mass induced by HS. However, in this study, Firmicutes and Bacteroides are predominant in the caecum of broilers at the phylum level, and the treatment of NAC failed to alter the composition of microbiota. In accordance with this finding in pigs, a similar abundance of cecal microbiota was noticed between the HS and the NAC groups [67], implying a limited effect of NAC treatment on the composition of the bacterial community in heat-stressed broilers. As one of consequences, the gut microbial metabolites, SCFAs, are well-established to exert a crucial role in gut development and bone remodeling. Drinking SCFA addition promoted intestinal development and tibia quality of ducks [27]. Serving as the fuel for intestinal epithelial cells (IEC) and directly regulating IEC functions, especially butyrate [68], thus the parallel levels of SCFAs might be linked to the comparable villus height and crypt depth of ileum in this study. Furthermore, the similar cecal microbiota composition and SCFAs content in cecum indicated that the attenuated roles exerted by dietary NAC inclusion is irrelevant to cecal microbiota in heat-stressed broilers.

## Conclusion

In the present study, implementing chronic HS evoked ROS excessive production and oxidant stress, resulting in compromised tibia mass. NAC supplementation in the diet of heat-stressed broilers exerted no effects in growth performance and microbiome, but it enhanced barrier function and antioxidant capacity of both the ileum and bone marrow. Moreover, the diet containing NAC increased tibia mass and improved bone microstructure via the restoration of the antioxidant defense and suppression of bone resorption under heat exposure conditions. The protective effect of NAC is likely associated with the activation of the Nrf2/NF-κB signaling pathway. The results also hint that the treatment of NAC might be a supplemental therapeutic for the prevention of intestinal disorder and bone loss induced by chronic HS in practice.

## Supplementary Information

Additional file 1: Table S1. Primers for quantitative real-time PCR. Fig. S1. Chemical structure of *N*-acetyl-L-cysteine and the temperature and relative humidity in heat stress room throughout the trial. Fig. S2. Verification of transcriptome analysis (RNA-seq) and RT-PCR. Fig. S3. Effect of dietary NAC in heat-stressed broilers on body weight, weight gain, feed intake, the ratio of feed consumption to body gain (F:G), and survival proportion during d 21 to 35.

## Data Availability

Data will be made available on request.

## Abbreviations

ALP: Alkaline phosphatase
FI: Feed intake
GSH-Px: Glutathione peroxidase
GST: Glutathione-*S*-transferases
HS: Heat stress
HSP: Heat shock protein
IL: Interleukin
MDA: Malondialdehyde
NAC: *N*-Acetylcysteine
Nrf2: Nuclear factor erythroid 2-related factor 2
RANKL: Receptor activator of nuclear factor-κ B ligand
ROS: Reactive oxygen species
SOD: Superoxide dismutase
TNF-α: Tumor necrosis factor alpha
TRAP: Tartrate-resistant acid phosphatase
ZO: Zonula occludens
