# Supplementary material for: Improvement of antioxidant capability by dietary N-acetyl cysteine supplementation alleviates bone loss induced by chronic heat stress in finisher broilers
Source: J Anim Sci Biotechnol. 2024 Dec 1;15:158. doi: 10.1186/s40104-024-01114-4 (PMC11608502; doi:10.1186/s40104-024-01114-4)
Supplement: Supplementary file 1 — Additional file 1: Table S1. Primers for quantitative real-time PCR. Fig. S1. Chemical structure of N-acetyl-L-cysteine and the temperature and relative humidity in heat stress room throughout the trial. Fig. S2. Verification of transcriptome analysis (RNA-seq) and RT-PCR. Fig. S3. Effect of dietary NAC in heat-stressed broilers on body weight, weight gain, feed intake, the ratio of feed consumption to body gain (F:G), and survival proportion during d 21 to 35. [file 40104_2024_1114_MOESM1_ESM.docx]

**Improvement of antioxidant capability by dietary *N*-acetyl cysteine supplementation alleviates bone loss induced by chronic heat stress in finisher broilers**

Huaiyong Zhang^1,2*^, Herinda Pertiwi^1^, Joris Michiels^1^, Djoere Gaublomme^3^, Maryam Majdeddin^1^, Yuhuang Hou^1^, Matthieu Boone^4,5^, Dirk Elewaut^3^, Iván Josipovic^4^ and Jeroen Degroote^1^

^1^ Laboratory for Animal Nutrition and Animal Product Quality, Department of Animal Sciences and Aquatic Ecology, Ghent University, Ghent 9000, Belgium

^2^ College of Animal Science and Technology, Henan Agricultural University, Zhengzhou 450046, Henan, China.

^3^ Unit Molecular Immunology and Inflammation, VIB Center for Inflammation Research, Ghent University and Department of Rheumatology, Ghent University Hospital, Ghent 9000, Belgium

^4^ Ghent University Centre for X-ray Tomography (UGCT), Ghent University, Ghent 9000, Belgium

^5^ Radiation Physics Research Group, Department of Physics and Astronomy, Ghent University, Ghent 9000, Belgium

* Corresponding author: Huaiyong Zhang

E-mail address: Huaiyong.zhang@ugent.be

**Table S1** Primers for quantitative real-time PCR

| **Gene** | **Gene ID** | **Primer** | **Sequence (5′-3′)** | **Size, bp** |
| --- | --- | --- | --- | --- |
| *GST* | NM_205365.2 | Reverse | cggagacttcaccctagcag | 138 |
|  |  | Forward | ttagcatgatgccacgagag |  |
| *CAT* | NM_001031215.2 | Reverse | ccacgtggacctcttcttgt | 95 |
|  |  | Forward | ttcgcatgcacaactctttc |  |
| *GPx1* | NM_001277853.3 | Reverse | atgttcgagaagtgcgaggt | 122 |
|  |  | Forward | atgatgtactgcgggttggt |  |
| *GPx2* | NM_001277854.3 | Reverse | atcgccaagtccttctacga | 141 |
|  |  | Forward | gagctgggtgtaatccctca |  |
| *GPx3* | NM_001163232.3 | Reverse | tcttccagaaaggggatgtg | 140 |
|  |  | Forward | tttgatgtcgtggttcctca |  |
| *SOD2* | NM_204211.2 | Reverse | gccacctacgtgaacaacct | 125 |
|  |  | Forward | ttgatatgacccccaccatt |  |
| *SOD1* | NM_205064.2 | Reverse | cttcaatcctgaaggcaagc | 87 |
|  |  | Forward | cctccctttgcagtcacatt |  |
| *HO-1* | NM_205344.2 | Reverse | catgcctacacccgctattt | 104 |
|  |  | Forward | aaagccaacccttctccagt |  |
| *Nrf2* | NM_001396902.1 | Reverse | aaaacgctgaaccaccaatc | 136 |
|  |  | Forward | gctggagaagcctcattgtc |  |
| *Claudin-1* | NM_001013611.2 | Reverse | gtctttggtggcgtgatctt | 117 |
|  |  | Forward | tctggtgttaacgggtgtga |  |
| *ZO-1* | XM_015278981.2 | Reverse | ggtcagccagatgtggattt | 81 |
|  |  | Forward | ccgaagcattccatcttcat |  |
| *Mucin-2* | NM_001318434.1 | Reverse | tgccagcctttttatgctct | 80 |
|  |  | Forward | agtggccatggtttcttgtc |  |
| *IL-1β* | NM_204524.1 | Reverse | gtttttgagcccgtcacct | 117 |
|  |  | Forward | cacgaagcacttctggttga |  |
| *IL-6* | NM_204628.1 | Reverse | ctcctcgccaatctgaagtc | 100 |
|  |  | Forward | ccctcacggtcttctccata |  |
| *TNF-α* | NM_204267.1 | Reverse | agatgggaagggaatgaacc | 120 |
|  |  | Forward | actgggcggtcatagaacag |  |
| *TGF-1β* | NM_001318456.1 | Reverse | ctgtacaacagcacccagga | 88 |
|  |  | Forward | gctctttggcccaatactca |  |
| *TLR4* | NM_001030693.2 | Reverse | tgcctgacagtctggtcttg | 108 |
|  |  | Forward | tccgcagtagatcctgcttt |  |
| *IkBa* | NM_001001472.3 | Reverse | ggggcagatgtgaacaaagt | 120 |
|  |  | Forward | tatctgcaggtcagctgtgg |  |
| *NF-kB p65* | NM_001396038.1 | Reverse | tctcccagcccatctatgac | 125 |
|  |  | Forward | ctggaccttgtcacagagca |  |
| *NLRP3* | NM_001348947.2 | Reverse | acagctcctctccctcttcc | 148 |
|  |  | Forward | gtcactgaccattccgtcct |  |
| *IL-10* | NM_001004414.4 | Reverse | ggagctgagggtgaagtttg | 147 |
|  |  | Forward | tagaagcgcagcatctctga |  |
| *INF-γ* | NM_205149.2 | Reverse | cagatgtagctgacggtgga | 98 |
|  |  | Forward | catcgaaacaatctggctca |  |
| *Runx2* | NM_204128.1 | Reverse | caggcatgtcactgggtatg | 115 |
|  |  | Forward | tatggagtgctgctggtctg |  |

| **Gene** | **Gene ID** | **Primer** | **Sequence (5′-3′)** | **Size, bp** |
| --- | --- | --- | --- | --- |
| *Osterix* | XM_015300329.3 | Reverse | gttcgtctgcaattggctct | 105 |
|  |  | Forward | agcacgcacgtgaatttctt |  |
| *BMP2* | XM_046938393.1 | Reverse | gccagaaacaagtgggaaaa | 101 |
|  |  | Forward | cccgaaaaatctggagttca |  |
| *Col1a1* | NM_001396622.1 | Reverse | ctgaagaaggctctgctgct | 116 |
|  |  | Forward | catgctccagtgtgactcgt |  |
| *Phex* | NM_001199277.2 | Reverse | tgccaactatctggtgtgga | 100 |
|  |  | Forward | tccatggatcactcgtgaaa |  |
| *Sost* | XM_025144077.1 | Reverse | gacagaaatcatccccgaga | 83 |
|  |  | Forward | cctggttcatcgtgttgttg |  |
| *Cathepsin K* | NM_204971.2 | Reverse | atatgaccagcgaggaggtg | 88 |
|  |  | Forward | gggacgtacagagtgccatt |  |
| *V-ATPase* | NM_001293241.1 | Reverse | ggcttggtgaagaaatccaa | 97 |
|  |  | Forward | accgagcagtttccatgttc |  |
| *OPG* | XM_015283019.2 | Reverse | tgggacaaagatcagcacag | 106 |
|  |  | Forward | ctccttgttttgctgcttcc |  |
| *RANKL* | NM_001083361.1 | Reverse | gtccagcgtattctgggaaa | 116 |
|  |  | Forward | atgagatgggcaaaaggttg |  |
| *NFATC1* | XM_015275940.4 | Reverse | agcgtcttcctacgagtcca | 111 |
|  |  | Forward | tggtacccctcctctgtgtc |  |
| *TRAF6* | XM_046942060.1 | Reverse | atgcaggtcacaaatgtcca | 126 |
|  |  | Forward | tcaaacagcagcctttgttg |  |
| *MMP9* | NM_204667.2 | Reverse | ccaagatgtgctcaccaaga | 108 |
|  |  | Forward | ccaatgcccaacttctcaat |  |
| *DKK2* | XM_046940772.1 | Reverse | gaagttgggcggtactgtca | 120 |
|  |  | Forward | gttgttgcagcgattcccag |  |
| *ETV4* | XM_015299450.4 | Reverse | caaccacaagcagccattcc | 112 |
|  |  | Forward | gattggggttcctgggtcag |  |
| *RAI2* | XM_015272982.4 | Reverse | ctgttgtcctccccatccac | 91 |
|  |  | Forward | tgtgtggtcatgacgtacgg |  |
| *FOXC2* | NM_001396046.1 | Reverse | cccgacaagaagatcacgct | 107 |
|  |  | Forward | cccgacaagaagatcacgct |  |
| *β-actin* | NM_205518.1 | Reverse | gctacagcttcaccaccaca | 90 |
|  |  | Forward | tctcctgctcgaaatccagt |  |
| *GAPDH* | NM_204305.1 | Reverse | tgggaagcttactggaatgg | 88 |
|  |  | Forward | cttggctggtttctccagac |  |

GST, glutathione S-transferase theta 1; CAT, catalase; GPx, glutathione peroxidase; SOD, superoxide dismutase; HO-1, heme oxygenase 1; Nfr2, nuclear factor, erythroid 2 like 2; ZO-1, zonula occludens-1; TRL4, toll like receptor 4; NF-κB, nuclear factor-kappa B; IκBa, IkB alpha; IL, interleukin; NLR family pyrin domain containing 3 (NLRP3); IFN-γ, interferon gamma; TNF-α, tumor necrosis factor alpha; TGF-β, transforming growth factor beta; Runx2, runt related transcription factor 2; Phex, phosphate regulating endopeptidase homolog x-linked; Sost, Sclerostin; V-ATPase, V-type proton ATPase; OPG, osteoprotegerin; RANKL, Receptor activator of nuclear factor-κ B ligand; BMP, bone morphogenetic protein; Col1a1, collagen type I alpha 1 chain; NFATC1, nuclear factor of activated T-cells 1; TRAF6, TNF receptor associated factor 6; MMP9, matrix metallopeptidase 9; DKK2, dickkopf WNT signaling pathway inhibitor 2; ETV4, ETS variant 4; RAI2, retinoic acid induced 2; FOXC2, forkhead box C2; GAPDH, glyceraldehyde-3-phosphate dehydrogenase


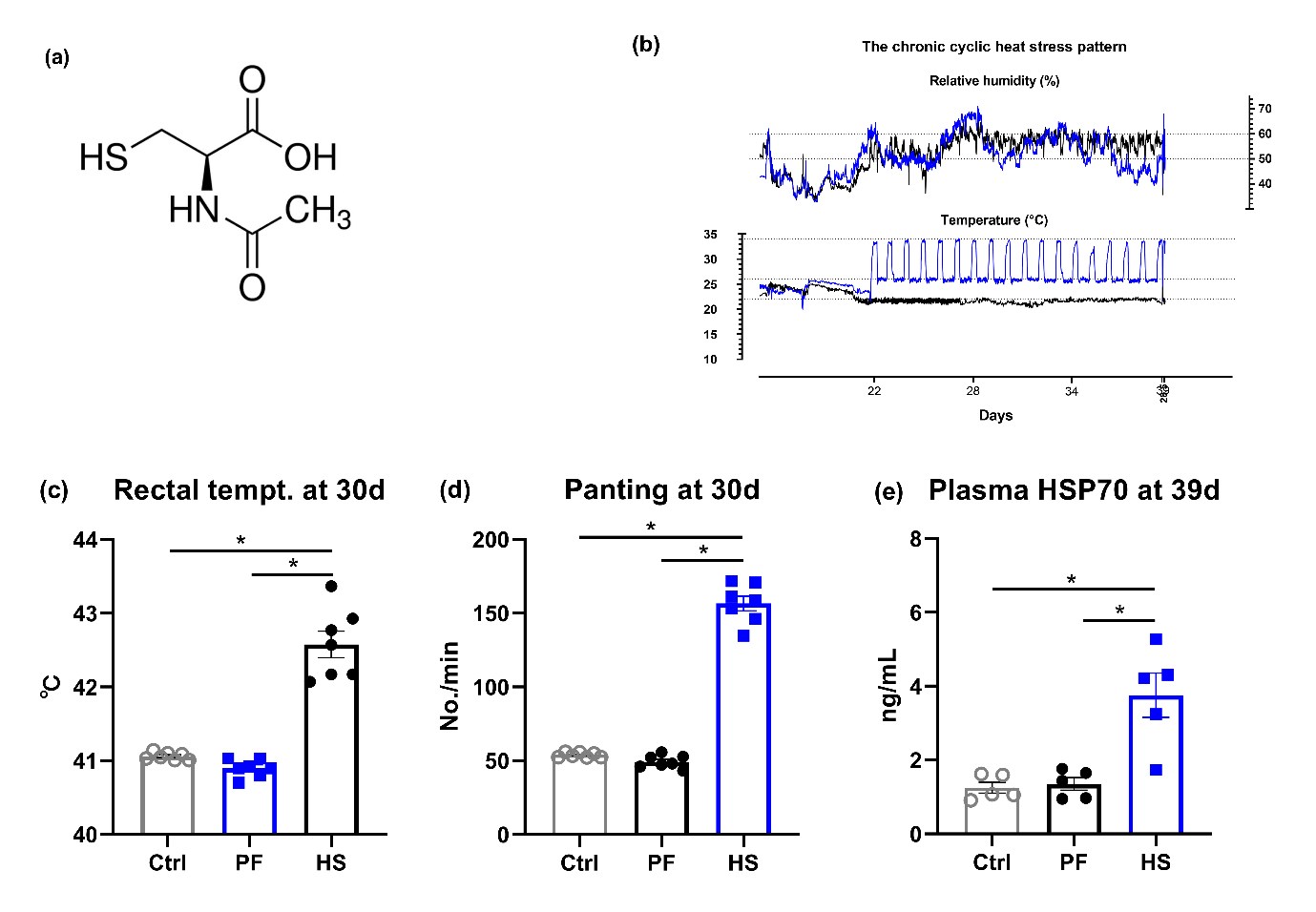


**Fig. S1** Chemical structure of *N*-acetyl-L-cysteine (**a**) and the temperature and relative humidity in heat stress room throughout the trial (**b**). The effect of HS on (**c**) rectal temperature, (**d**) panting, and (**e**) plasma heat shock protein 70 (HSP70). * Denotes significant difference among Ctrl, PF, and HS at *P* ≤ 0.05


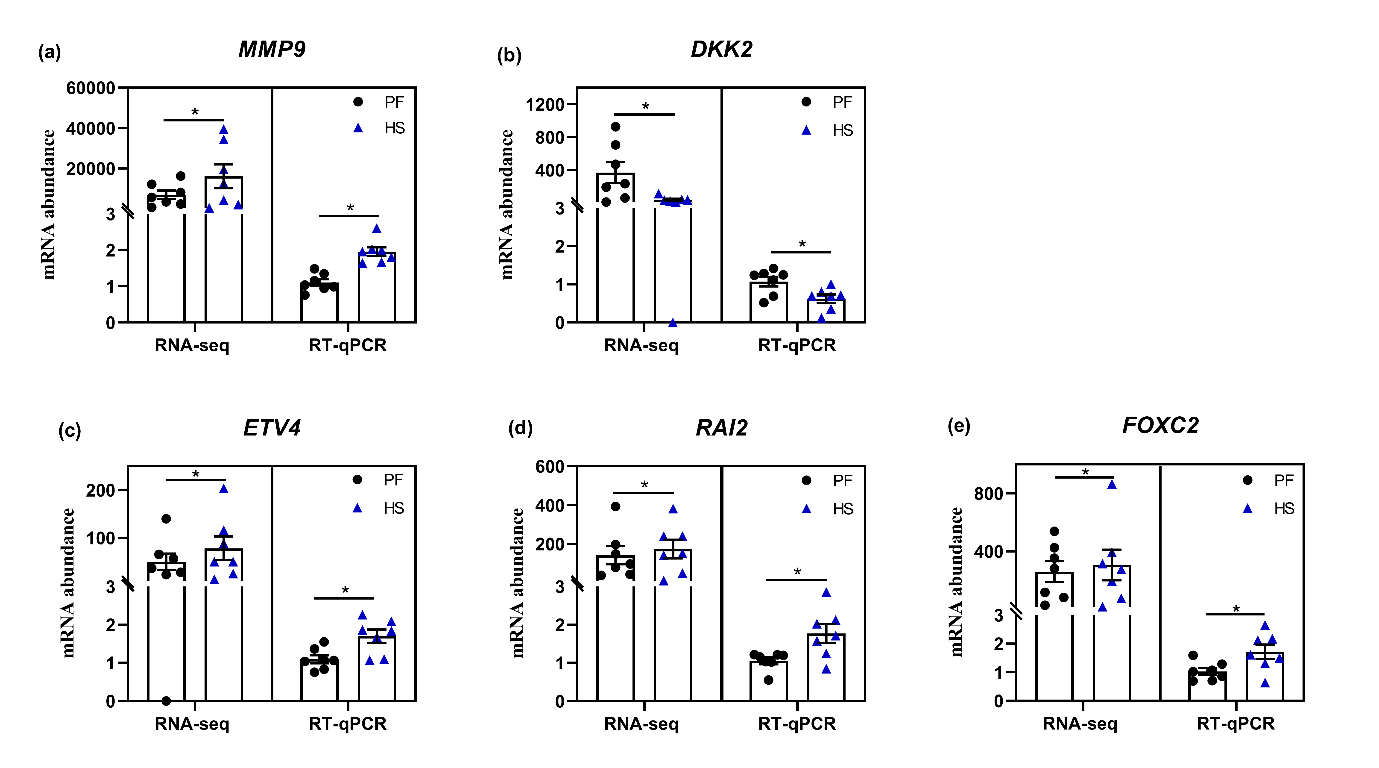


**Fig. S2** Verification of transcriptome analysis (RNA-seq) and RT-PCR using matrix metallopeptidase 9 (*MMP9*), dickkopf WNT signaling pathway inhibitor 2 (*DKK2*), ETS variant 4 (*ETV4*), retinoic acid induced 2 (*RAI2*), and forkhead box C2 (*FOXC2*). All the results were shown as mean ± standard deviation. An unpaired two-tailed *t*-test analysis was used to evaluate differences at *P* ≤ 0.05


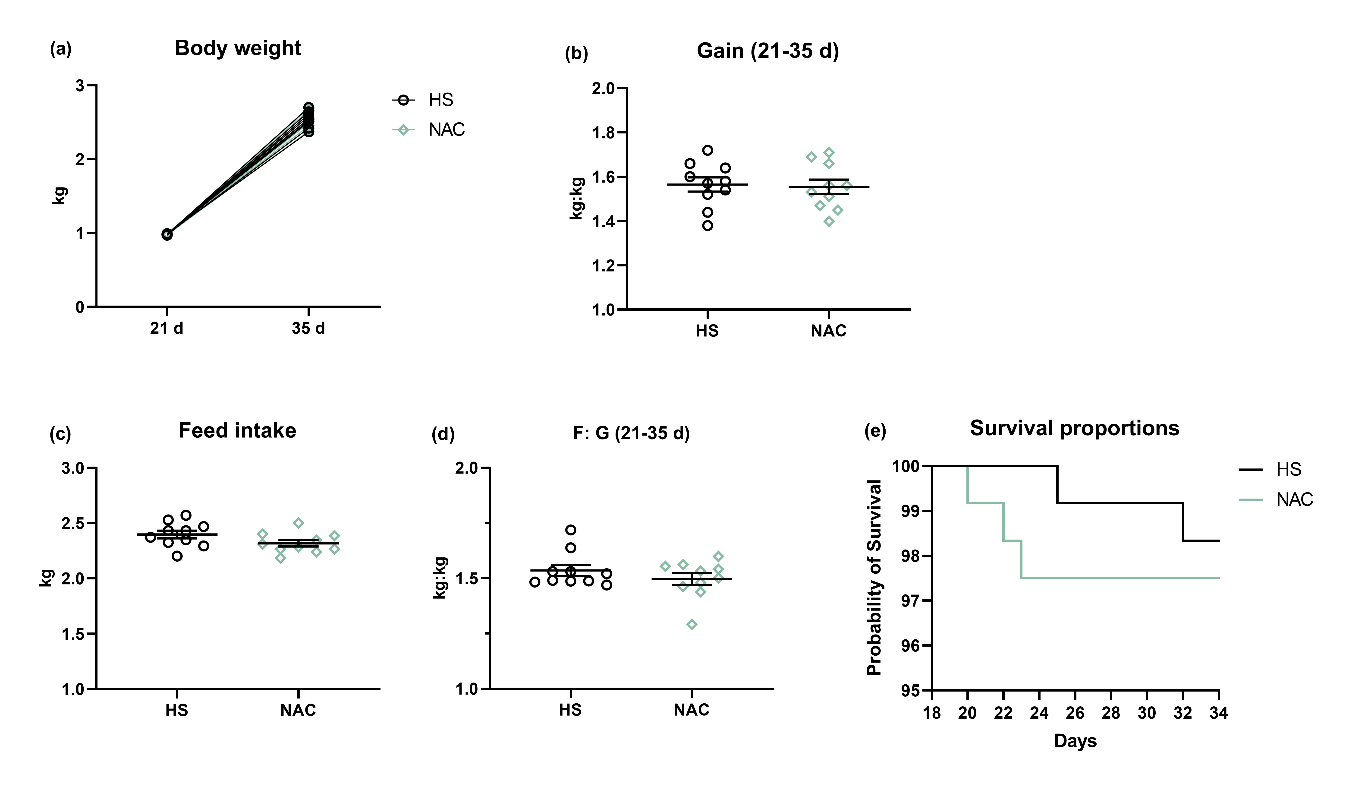


**Fig. S3.** Effect of dietary NAC in heat-stressed broilers on (**a**) body weight, (**b**) weight gain, (**c**) feed intake, (**d**) the ratio of feed consumption to body gain (F:G), and (**e**) survival proportion during d 21 to 35. All the results were shown as mean ± standard deviation. An unpaired two-tailed *t*-test analysis was used to evaluate differences at *P* ≤ 0.05. Survival analysis was performed using the Kaplan-Meier method
